# Reimagining public–private partnerships for primary health care in Africa: a governance and systems analysis of emerging models in Botswana

**DOI:** 10.3389/fpubh.2026.1862905

**Published:** 2026-07-13

**Authors:** Thabo Lucas Seleke

**Affiliations:** Political Science and Public Administration, University of Botswana, Gaborone, Botswana

**Keywords:** Botswana, decentralization, health systems governance, primary health care, public–private partnerships, stewardship, strategic purchasing

## Abstract

**Background:**

Primary health care (PHC) systems across many low- and middle-income countries increasingly rely on mixed provider environments involving public, private, and mission-based actors. Public–private partnerships (PPPs) are often promoted as a way of improving coordination and expanding access to care. However, many PHC partnerships continue to be governed through highly centralized and compliance-oriented arrangements that may limit flexibility, trust, and long-term collaboration. Botswana provides a useful case for examining how PPPs are governed within a health system experiencing fiscal pressure, medicines supply challenges and ongoing PHC restructuring.

**Methods:**

This study relies on a theory-based qualitative documentary analysis of policy documents, government reports, gray literature, and peer-reviewed research relevant to PHC governance and PPP implementation in Botswana and comparable low- and middle-income country settings. Concepts such as health systems governance, stewardship and systems thinking guided the analysis with due attention to financing arrangements, accountability systems, provider relationships and implementation dynamics within mixed health systems.

**Results:**

Four recurring governance barriers were identified as factors affecting PPP implementation in PHC. These included hierarchical administrative approaches that limit collaboration and local adaptation; weak financing credibility and purchasing uncertainty; limited community-facing accountability; and gaps between national policy ambition and district-level implementation capacity. The analysis also identified emerging opportunities related to digital health systems, integrated service delivery approaches, and more flexible partnership practices that may strengthen coordination across providers.

**Conclusion:**

The findings suggest that PPP performance in PHC depends less on formal contracts alone and more on the wider governance conditions shaping relationships between public institutions, providers, and communities. In Botswana, partnership arrangements are more likely to function effectively when financing systems are credible, accountability extends beyond administrative reporting, and local implementation systems are adequately supported. The study contributes to wider debates on PHC governance by highlighting the importance of stewardship, coordination, and institutional trust in sustaining partnerships within mixed health systems.

## Introduction

Many low- and middle-income countries face increasing pressure on primary health care (PHC) systems due to population growth, urbanization, rising burdens of chronic disease, and continuing infectious disease challenges. Governments are expected to deliver more services despite constrained resources, uneven infrastructure, workforce shortages, and fragmented programmes (1–4). As global attention has increasingly focused on universal health coverage, PHC has been reaffirmed as the foundation of health system performance and service delivery.

PHC increasingly operates within mixed provider environments. Patients seek care at government facilities, private practitioners, pharmacies, laboratories, faith-based organizations and digital platforms based on cost, availability, trust and convenience (5, 6, 51). Public–private partnerships (PPPs) have consequently emerged as one mechanism for coordinating service delivery across these diverse actors.

However, PHC does not fit neatly within traditional PPP models developed for infrastructure, procurement, or tightly controlled contracting arrangements. Although governance arrangements that emphasize compliance may improve administrative control, they may also generate frictions in service milieus that are based on trust, responsiveness and continuous collaboration of providers (7–9, 48). The central question is therefore not whether private actors participate in PHC, but how their participation is governed within wider health systems.

Botswana provides a useful case through which to examine these issues. The country has historically invested heavily in public health and achieved notable gains in HIV treatment and health programming. More recently, however, concerns relating to medicines availability, procurement delays, stock management, and broader system coordination have received increased attention. Similar concerns have appeared periodically during earlier reforms of the Central Medical Stores (CMS) and have also been highlighted in audit reports examining medicines management and oversight systems (10, 11).

At the same time, Botswana has undergone significant institutional restructuring within PHC governance. In 2010, responsibility for PHC services shifted from local authorities to the Ministry of Health. More recently, Government Notice No. As part of a wide restructuring, in 2024, clinics, community-based services and public health functions were transferred to the Ministry of Local Government and Traditional Affairs (12). By mid-2025, hundreds of facilities, district health management offices, staff houses and operational assets across the country were transferred (13). These developments have rekindled questions of coordination, accountability, financing responsibilities and district-level implementation capacity.

Against this background, this study examines PPPs for PHC in Botswana through a theory-informed qualitative governance analysis using documentary and scholarly sources. Rather than treating PPPs primarily as technical contracting instruments, the analysis approaches them as arrangements shaped by financing credibility, accountability structures, administrative relationships, and implementation capacity within a plural health system (14, 15). Botswana is therefore used not as a representative national model, but as an exploratory governance case through which wider questions concerning PHC partnerships, decentralization, and health system coordination can be examined under conditions of fiscal pressure and institutional change.

The paper contributes to debates on PHC governance by arguing that partnership performance is shaped not only by contractual arrangements but also by broader governance conditions, including institutional alignment, financing credibility, provider diversity, and accountability relationships. It highlights the critical nature of stewardship, coordination, and community accountability, and how public and private providers can achieve more together in the same health system.

## Conceptual framework: public–private partnerships, governance, and systems thinking

### From hierarchical contracting to partnership-led governance in primary health care

Public-private partnerships in health have often been framed through contracts. This is understandable. Governments need rules, standards, payment controls, and ways of holding service providers accountable. In many health systems, especially where public funds are involved, these controls are not optional. They protect public resources and help guard against weak performance, unfair pricing, and unsatisfactory service (7).

The challenge lies with PHC not being a contracts issue only. There are relationships to consider. There is a need for PHC to rely on trust, continuity, referral networks, knowledge of the community, and daily coordination among the various players. A regulatory compliance approach may fail to capture this. It is likely to hold for infrastructural projects or hospital-related contracts. However, it is probably less applicable in the case of small clinics, local pharmacies, laboratories, and community-facing providers who are more peripheral and proximate to the day-to-day activities of the community (5, 8, 9).

This is a critical distinction when considering mixed health systems. In these systems, a larger private provider is not the only private provider. There are large, well-capitalized, and vertically integrated private providers, and there are small private providers with small capital and thin private, and often poor, administrative capacity. If all private providers are viewed as large, capitalized private providers, small private providers may not even be able to participate. The prospect of delayed payments with unclear lines of command, and increasingly costly reporting requirements, makes partnerships unattractive, despite the potential to enhance access to health care (5, 8).

A governance framework asks a different kind of question. It does not limit itself to the mere existence of a contract. It asks if the surrounding governance structures, to include the rules, the incentives, and the relationships, allow the partnership to function. This is the role of stewardship. While it provides a fit for purpose regulation, it does not eliminate the need for regulation. In this sense, it provides a balance of the control and regulation with the governance of trust and the public good (9, 48).

In this case, partnership-led governance refers to the structure beyond a collaborative approach. It denotes the arrangements where the public authorities, private providers, and the community are assigned clearer responsibilities and roles, as well as the opportunity to address and resolve issues pertaining to the execution of the arrangements.

This matters in PHC since service gaps can be local and practical as opposed to strictly contractual. When looking at Botswana, this perspective allows for the evaluation of why PPPs shouldn't be evaluated and concluded on the bases of formal policy. Commitments. Rather, PPPs should be evaluated and concluded on the bases of their financing, coordination, and accountability.

### Health systems as complex adaptive systems: implications for PPP design

When health systems are regarded as intricate and ever evolving systems, the limits of control-oriented PPP models become even more obvious. Health systems function in a messy and unpredictable manner. Actors have various methods of responding to rules, incentives, and pressures, and their methods can vary over time (15–17). As a result, policies that seem to be clear and straightforward can generate inconsistent outcomes when implemented in actual service environments.

This is applicable in the context of PPPs in PHC. It is true that contracts and reporting systems can provide some level of order and accountability, but they are not able to perfectly specify the answers that providers, managers, and communities will give. In certain contexts, enhanced coordination may be a result of controlled mechanisms, but in other contexts they can produce delays, irritation, and even, at a minimum, disengagement, particularly in the instances when service providers have been excluded from the overall decision-making and are unable to easily adjust to the control of the flexible mechanisms of administration (15, 17).

Studies about reform in health systems show, time and time again, that implementation is a result of the system's ability to learn and evolve (14, 16). Discrete policy design and the improvement of a problem because of that design will never be achieved because of the emergence of new problems and the changes in behavior, as a result of financial pressure on the systems. Governance frameworks that rely only on control may, therefore, struggle to respond when conditions begin to change.

Systems thinking has the advantage of focusing more on learning and the capacity to adapt and absorb feedback as opposed to rigid adherence to processes (14, 18). Regarding PPPs in PHC, this suggests the governance structures cannot be reliant on rigid contractual arrangements there are important roles for joint evaluation, ongoing dialogue, and mechanism adjustment. This is especially the case in PHC where care continuity, system responsiveness, and collaboration among care providers shape the health system experience for users.

### Decision science, trust, and institutional behavior in mixed health systems

In PHC, partnerships are not solely contractual. They are also shaped by how institutions and providers respond to uncertainty, stress, and risk. When financial or political pressures increase, public institutions may favor more centralized and tightly controlled arrangements because they provide a greater sense of predictability and control, even when such arrangements reduce flexibility (19, 20). Similar pressures affect providers. Smaller providers, in particular, may become more cautious when faced with payment delays, unstable operating environments, or uncertainty about long-term policy commitments (21).

In this context, trust becomes an important governance resource. It influences the ability of organizations to establish and sustain partnerships under conditions of uncertainty. PHC depends heavily on cooperation between providers, public institutions, and communities. When financing becomes unstable or policy environments become uncertain, trust may weaken, making partnerships more difficult to sustain (9, 22).

The implications for accountability are equally important. Sustained cooperation is more likely in systems that create opportunities for participation, communication, and community oversight (8, 23). In PHC, where services are used repeatedly over time, community confidence and patient feedback play an important role in shaping the legitimacy of both public and private providers. International PHC frameworks increasingly recognize community participation as an important component of accountability within mixed health systems (24, 25).

In this study, trust is understood as a governance issue. Partnership arrangements are more likely to function effectively when providers and communities perceive financing arrangements, rules, and institutional processes as fair, predictable, and supportive of long-term participation.

## Methods

### Study design and analytical orientation

This study examines the governance, framing, and implementation constraints of public–private partnerships (PPPs) for primary health care (PHC) in Botswana through a theory-informed qualitative governance case analysis. The analysis relies on documentary and scholarly sources and does not include interviews, survey data, or fieldwork. Botswana is treated as an exploratory governance case through which broader questions concerning PPP governance in mixed health systems can be examined.

This approach is appropriate because PPPs in PHC involve more than formal contracts and technical service arrangements. Partnership performance is also shaped by financing systems, coordination mechanisms, institutional relationships, trust, and implementation capacity, issues that may be less visible through contract-focused or quantitative approaches.

The study is neither a systematic review nor a scoping review. Rather, it adopts an interpretive documentary approach drawing on policy documents, government reports, gray literature, and peer-reviewed scholarship selected for their relevance to PPP governance and PHC reform.

The analysis is informed by approaches commonly used in health policy and systems research, which emphasize the influence of institutions, actors, and context on health system performance (14, 15). Document analysis is used not only as a source of information but also as a means of examining how governance challenges, policy assumptions, and institutional arrangements are represented within policy and organizational frameworks (26).

The focus is therefore on how PPP governance is articulated, justified, and operationalised within policy and institutional settings, with particular attention to governance arrangements, implementation constraints, and gaps between policy intentions and practice.

### Data sources and document selection

The analysis is based on policy documents, government reports, gray literature and peer-reviewed research on PPP governance and primary health care (PHC), with a particular focus on Botswana and similar low- and middle-income country (LMIC) contexts. Documents were selected purposively based on their relevance to PHC governance, financing arrangements, accountability systems, institutional coordination, and partnership implementation. The objective was not to review all available literature on PPPs or PHC, but to identify materials capable of informing the governance dynamics of partnership arrangements within mixed health systems.

The document corpus included national health policies, PHC reform documents, health financing and supply-chain reports, government publications, international policy frameworks, and peer-reviewed studies on PPPs, mixed health systems, and health governance. Particular attention was given to literature addressing decentralization, strategic purchasing, medicines supply systems, provider participation, and accountability arrangements.

Document identification was undertaken iteratively between January and April 2026 using Google Scholar, PubMed, WHO databases, government websites, and targeted searches of policy and institutional repositories. Search terms included combinations of “primary health care”, “public–private partnerships”, “health governance”, “strategic purchasing”, “Botswana”, “mixed health systems”, “decentralization”, and “medicines supply systems”. The final corpus included policy documents, government reports, global PHC frameworks, peer-reviewed journal articles, and selected gray literature.

Comparative literature from other LMICs was included to contextualize Botswana within broader discussions of provider engagement, accountability and the governance of partnerships. This literature was used thoughtfully, not to make direct comparisons between countries, but to identify common governance and implementation challenges in mixed health systems. The analysis was also informed by global PHC frameworks, including the Declaration of Astana and the WHO Operational Framework for Primary Health Care, which continue to shape contemporary discussions on people-centered care, community participation, multisectoral action and health system coordination (24, 25).

Because documentary evidence can present policy reforms in a more coherent manner than they are experienced in practice, official documents were interpreted alongside comparative scholarly literature and governance studies from other LMIC settings. This enabled a more critical engagement with policy claims, implementation gaps, and governance tensions across the material.

### Analytical framework and interpretive approach

The material was analyzed using a theory-informed thematic approach that moved iteratively between the governance literature and the Botswana case material. The analysis focused on how PPP governance is framed within policy and institutional documents, where implementation challenges emerge, and how these challenges relate to broader PHC reform processes.

The first stage applied governance concepts commonly used in health policy and systems research, including transparency, accountability, participation, integrity, institutional capacity, and coordination. These concepts were used to examine how PPP arrangements define roles, distribute authority, manage public resources, and establish mechanisms for oversight.

The second stage allowed themes to emerge inductively from the material. Within Botswana-focused documents, attention was given to recurring issues such as medicines supply pressures, procurement concerns, PHC restructuring, district-level capacity, and coordination between national and local institutions. Within the wider literature, attention focused on provider participation, payment credibility, administrative burden, and trust between public and private actors.

The third stage compared emerging themes with the conceptual framework developed earlier in the paper. Systems thinking was used to examine how financing, procurement, regulation, provider behavior, and implementation capacity interact within PHC systems over time (15–17). PPPs were therefore analyzed not as isolated contractual arrangements but as governance arrangements embedded within wider health systems.

Interpretation was further informed by institutional and political economy perspectives, particularly in explaining provider responses to uncertainty and the tendency toward greater administrative control during periods of systemic pressure (21). Behavioral perspectives were retained where relevant to understanding decision-making under uncertainty (19, 20).

The analysis was interpretive rather than causal and sought to identify recurring governance themes and patterns across the documentary and scholarly literature.

### Analytical purpose and contribution

The purpose of the analysis is interpretive rather than causal. The study does not seek to measure the effects of public-private partnerships (PPPs) on primary health care (PHC) outcomes or to generate findings that can be statistically generalized. Instead, it provides a governance-oriented account of how PPPs are situated, conceptualized, and articulated within an integrated health system context (14, 15). Consistent with health policy and systems research, the analysis emphasizes explanation, contextual interpretation, and an understanding of the complex institutional relationships that shape policy implementation.

The study examines developments in PHC reform in Botswana and situates them within wider international debates concerning partnership governance, accountability, and health-system integration. Drawing on both documentary and scholarly sources, the analysis focuses on governance conditions that influence implementation, including financing uncertainties, procurement arrangements, provider accountability, decentralization processes, coordination across service providers, and mechanisms of community accountability.

Rather than treating PPPs as stand-alone contractual arrangements, the study examines them as components of a broader PHC governance system embedded within existing institutional structures and health-system governance arrangements (7, 9). Botswana is therefore approached as an exploratory governance case rather than a representative case.

The value of the case lies not in its statistical representativeness but in its capacity to illuminate broader governance pressures affecting PHC reform in mixed health systems operating under conditions of fiscal and institutional stress. These pressures include tensions between central control and local responsiveness, formal policy commitments and operational realities, and partnership expansion and system readiness.

The findings are therefore presented as analytical propositions rather than definitive causal conclusions. They identify governance issues that may warrant deeper empirical investigation, including provider payment arrangements, contracting practices, district-level implementation capacity, medicines procurement systems, and community oversight mechanisms. In doing so, the paper contributes to ongoing debates within the health systems governance and PHC reform literature regarding how public and private actors can operate more coherently within a shared health system under conditions of uncertainty and institutional change (14, 24).

### Results: governance barriers and emerging opportunities

Across the documentary corpus and the comparative literature, Botswana's PHC PPP landscape appears best characterized as a mixed system with under-institutionalized partnership governance: public policy documents repeatedly signal an intention to “strategically purchase” and collaborate with non-state providers, however, the operational logic in contracting and regulation often retains control-oriented, compliance-heavy features that limit co-creation and learning (9, 27, 28). In practice, this creates predictable tensions for PHC partnerships, especially where the private sector is heterogeneous and includes small community-embedded providers (pharmacies, micro-clinics, maternity homes, laboratories) whose participation depends on credible payment arrangements, clear performance expectations, and manageable transaction costs (5, 8). The results are organized into four governance barriers and one set of emerging opportunities.

### Hierarchical regulation and constrained co-creation

One of the clearest patterns emerging from the documentary analysis is the continued reliance on hierarchical administrative approaches in the governance of PHC partnerships. Across the literature on PPPs and mixed health systems, heavily centralized arrangements are often associated with weak provider engagement, limited flexibility and strained operation relationships, mostly where contracting systems depend on rigid compliance procedures and standardized reporting demands that are weakly adapted to local conditions (5, 48). In these contexts, there could be formal partnerships, but opportunities for combined problem-solving and local adaptation are still limited.

This tension seems pertinent to Botswana's PHC environment. National policy documents back partnering with private and mission providers and contextualize partnership frameworks as creating integrated and universal service delivery (9, 27). However, the reviewed material also shows that procurement, regulation, and administrative procedures may create obstacles to partnership for smaller providers. Recent PHC restructuring and the transfer of services between ministries have also increased coordination demands across different levels of the health system, creating additional administrative requirements for partnership management (12, 13). Similar sentiments are expressed in the broader PPP governance literature where reporting, accreditation, and payment verification become burdensome for providers with limited administrative and financial resources (5, 8).

This is important because smaller, community-based, and peripheral PHC system providers such as pharmacies, laboratories, maternity services, and clinics are the cornerstone of many health systems. These providers work with smaller financial margins, and are more susceptible to delays, uncertainties, and administrative costs. If partnership systems are restrictive, and therefore not beneficial, providers are likely to withdraw from the system and/or avoid entering formal contractual arrangements (8, 48). Instead of augmenting integration, governance systems inadvertently increase fragmentation of the health system by reinforcing the divide between public and private sectors.

Literature on comparative governance corroborates this account. Studies focusing on PPP governance in PHC show that participation, relationship management, and communication facilitate successful partnerships. In contrast, top-down allocation systems and enigmatic decision-making tend to yield poor implementation results (5). In the case of Botswana, policy advocacy for the expansion of partnerships may not be adequate in governing relationships. The management of relationships, including the governance and oversight of service providers, may be as important as the existence of partnership agreements (8, 9, 28).

### Financial misalignment, risk asymmetry and credibility deficits

A new governance barrier identified from the analysis is the relationship between public purchasing systems and the financing realities of private PHC providers. In mixed health systems, partnerships become operational when providers regard public purchasing systems to be real in the form of payment systems that are made in a timely manner, and reimbursement rules that allow planning and administration without uncertainty about claims and contracts (21, 28). In the absence of the conditions mentioned, providers will respond by offering fewer services, limiting financial risk, or withdrawing from partnerships (21).

This problem seems more and more pertinent to Botswana's PHC ambience. The documentary material examined shows extensive fiscal pressures targeting certain health sector areas, notably the procurement and stability of medicine supply systems. It is fair to say that these pressures go beyond PPP. However, they illustrate, along the same continuum, a governance issue that the international PPP literature also identifies when financing systems are regarded as slow, unpredictable, or difficult to navigate, partnerships are hard to sustain (27, 28). In these cases, the uncertainty of purchasing and reimbursement will dictate provider behavior long before contracts are formally terminated.

Recent developments in Botswana's health system help illustrate these pressures more clearly. Delays in procurement, medicines shortages, and supply chain instability have generated renewed attention to the credibility and coordination of public purchasing systems. These concerns are not entirely new. Earlier CMS reform documents identified weaknesses relating to communication, management, and supply chain visibility (10). Similarly, the Auditor General highlighted concerns relating to stock management, expired medicines, and inefficiencies within medicines management systems (11). More recently, the 2026 Forensic Audit Summary Report identified medical procurement and supply chain management among the areas requiring further institutional scrutiny and strengthening (29). Together, these developments suggest that procurement governance and financing credibility remain important considerations for the sustainability of PHC partnerships.

Many studies that focus on purchasing reforms and results-based financing in LMIC contexts support this perspective. Meessen et al. (30) suggest that payment reform alone does not resolve implementation challenges, including poorly aligned financing gaps, provider autonomy, and verification systems. Local level implementation of purchasing reforms may be hampered by delayed disbursements, reduced decision-making, and slow administrative processes. This is especially relevant in a Primary Health Care (PHC) context, as are smaller providers who often have limited financial reserves and risk cash flow issues.

The Botswana experience suggests that expanding contractual arrangements alone may not strengthen collaboration if underlying governance relationships between public and private actors remain weak. The purchasing system's reliability is critical. Delays in payment, uncertainty about reimbursement, and unpredictable budgets during fiscal constraints may erode confidence and provider participation in the long term (21, 28, 30).

### Weak community participation and downstream accountability

The third governance barrier that the analysis presents relates to the lack of community involvement and downstream accountability in PHC partnerships. PHC partnership arrangements are based on close contact between the service provider, the user, and the community. When partnerships are primarily controlled through central reporting and compliance, the accountability is more likely to be directed to the buyers and regulators and less likely to be directed to the community (22, 23). In these cases, concerns on the service experience and provider responsiveness may be addressed only after meeting the administrative reporting needs.

The comparative literature on social accountability provides useful insight here. In several African settings, the community structures and health facility committees strengthened accountability and improved service delivery, including transparency concerning medicines and staffing, as well as the resolution of local problems (23).

At the same time, the literature also shows that these structures do not function automatically. Their effectiveness often depends on whether they have clear responsibilities, community legitimacy and meaningful links to formal decision-making systems.

Many studies related to the Community Score Card and community–provider engagement systems demonstrate that some PHC systems exhibit improved communication and greater trust and responsiveness in the community (31, 32, 49). However, challenges persist, especially about the representation of marginalized groups and local voices and priority's ability to influence decision making beyond the facility.

This issue is significant for Public Private Partnerships (PPPs) because private providers often expand first in areas where public systems are under strain, such as medicines, diagnostics, after-hours care, and smaller specialized services. Future partnership agreements may unintentionally increase existing inequalities in the absence of stronger community-facing accountability. Communities that have a stronger voice, more and better information, or more regional influence will have better and more system responsiveness, while the more vulnerable and remote will not be seen by the system (23, 31, 49).

For Botswana, the analysis suggests that community participation may need to be treated as part of partnership governance rather than as a separate consultation exercise. Current PHC reform ambitions increasingly emphasize integrated and people-centered service delivery across public and private settings (25, 28). Under these conditions, accountability systems that create stronger links between providers, communities and local decision-making structures may become increasingly important for sustaining trust and responsiveness within mixed PHC systems.

### National–subnational disconnect and implementation capacity constraints

The fourth governance barrier concerning the analysis relates to the disconnect between what the national policy would want to achieve and what is realistically possible at the local level. In many of the mixed health systems, policy-level partnership reforms look coherent and manageable, but they become much more challenging at the district and facility levels, where the burdens of implementation are felt the most (33, 34). In PHC, partnership reforms typically expect local managers to do the coordinating, monitoring, and problem resolution while also assuring the continuity of care but do so with limited resources.

It has been widely noted that centrally placed reforms will not seamlessly integrate across multiple local settings. Outcomes are shaped by local relationships, institutional capacity, information flows and resource availability, all of which vary across districts and service environments (14–16). These pressures become even more visible in PHC systems because services are geographically dispersed and often depend on coordination across multiple facilities, providers and administrative levels.

Botswana's PHC policy direction continues to support decentralized planning and district-level implementation aligned with national priorities (27). Parliamentary and government reports indicate that the transfer involved clinics, health posts, district health management offices, staff houses and other operational assets across the country. The scale of the transition has reinforced the importance of coordination between national and local institutions, particularly where service delivery responsibilities, resources and accountability arrangements continue to evolve (12, 13). However, comparative decentralization literature suggests that reforms may struggle where responsibilities are transferred without corresponding decision-making authority, financial flexibility or operational support at local level (33, 34). Similar patterns have been reported in decentralized PHC reforms elsewhere in the region.

In some settings, local systems became more responsive after reforms were introduced. However, implementation problems did not disappear completely. In many settings, funding pressures remained a challenge even after reforms had started. Moghadam et al. (35) further note that routine financial pressures, human resource constraints and weak financial management continued to slow implementation in some decentralized PHC settings.

These pressures are also felt locally in respect of PPPs. Arrangements that seem clear at policy level may become difficult to run once districts and facilities are expected to implement them. Procedural inconsistencies and variations in administration can erode trust and engagement from providers (5, 33). The analysis further illustrates that some local innovations may be unable to progress from isolated innovations in the absence of the capacity to link, integrate, and manage contracts, referral pathways, supervision, and reporting among the various providers and levels of care in a district.

In the current context of Primary Health Care (PHC) in Botswana, these implementation pressures may become more visible as PHC responsibilities continue to be redistributed across national and local government structures. The analysis therefore suggests that PPP governance cannot be understood only at national policy level. The ability of districts and local systems to coordinate relationships, manage routine implementation pressures and sustain provider engagement also appears central to whether partnerships function effectively in practice (14, 16, 35).

### Emerging opportunities: digitalisation, flexible partnership practices and quality infrastructures

Despite the governance pressures identified above, the analysis also highlights opportunities for developing more coordinated and responsive PHC partnerships in Botswana. These opportunities point toward governance arrangements that place greater emphasis on coordination, learning and system responsiveness rather than tightly controlled partnership management.

One opportunity lies in the continued development of digital health systems. Botswana's policy direction already signals growing interest in strengthening digital health infrastructure and improving the use of information systems within the health sector (36). Across many LMIC settings, digital systems are increasingly discussed not only as clinical tools, but also as mechanisms that can support coordination, continuity of care, purchasing oversight and communication across providers (37, 50). At the same time, the literature cautions against treating technology as a standalone solution. Digital systems are more likely to succeed when embedded within existing service structures and supported by strong governance arrangements, staff capacity and political commitment (38, 50).

The analysis also highlights the importance of more adaptable partnership approaches. The comparative PPP literature increasingly suggests that partnerships are more effective when supported by clear governance arrangements, open communication and predictable monitoring systems, together with stronger relationship management among key actors (5, 39). This does not imply the absence of regulation. Rather, it suggests that partnership arrangements may function better when accountability requirements are balanced with operational flexibility and local adaptation. In PHC settings, where providers operate under different conditions and capacities, rigid uniform approaches may not always produce the intended outcomes.

A third opportunity concerns the development of shared quality and coordination structures across public and private providers. Botswana's broader PHC policy direction continues to emphasize integrated and people-centered service delivery involving public institutions, mission facilities and private actors (9, 28). This creates space for approaches that strengthen referral coordination, supervision, reporting standards and continuity of care across different provider types rather than maintaining fragmented parallel systems.

Overall, the analysis suggests that future PPP development in Botswana may depend less on expanding partnerships alone and more on how governance systems support coordination, trust and practical problem-solving across institutions. Digital systems, purchasing reforms and quality improvement structures may all contribute positively, but their impact is likely to depend on the broader governance environment within which partnerships operate (21, 23, 39).

## Discussion

### Reframing PPPs for PHC: from contractual control to stewardship and co-creation

The findings from Botswana point toward a broader governance issue that also appears across many mixed health systems: PHC partnerships are difficult to sustain through contractual control alone. While compliance-oriented arrangements are often introduced to strengthen accountability and protect public resources, the Botswana findings suggest that partnership performance may depend not only on formal contractual arrangements but also on the ability of governance systems to support coordination, communication, and adaptation in routine service delivery (5, 8, 39). This may be particularly important for smaller providers operating with limited administrative and financial resources.

This aligns with broader governance literature, particularly debates on stewardship versus command-and-control regulation, which emphasize the importance of coordination, negotiation, and relationship management alongside formal accountability mechanisms (9, 48). In PHC systems, where trust, responsiveness, and continuity of care are central, effective partnership governance is likely to depend on the ability of organizations to build and sustain relationships across different actors and levels of the health system.

The analysis also acknowledges the importance of regulation and contractual accountability. Regulatory frameworks remain essential for safeguarding equity and maintaining minimum service standards. However, the Botswana case suggests that PPPs may struggle where governance systems rely predominantly on administrative control while giving less attention to communication, coordination, and operational problem-solving. Similar concerns have been raised in the broader PPP governance literature examining PHC reforms in LMIC settings (7, 8).

The findings suggest that PPPs in PHC are unlikely to achieve their intended objectives when viewed solely as procurement arrangements. Rather, they may function more effectively where health systems are stable, local structures have the capacity to implement partnerships, and providers, communities, and public institutions are able to develop and sustain relationships of trust (9).

Taken together, these findings support a broader shift in how PPPs in PHC are understood. Rather than viewing partnerships primarily as procurement or contracting mechanisms, the Botswana case suggests that their effectiveness depends on wider governance conditions, including institutional trust, coordination, communication, and implementation capacity. From this perspective, stewardship is not an alternative to accountability, but a complementary approach that helps partnerships function more effectively within complex and evolving health systems.

### Financial credibility and strategic purchasing as foundations of partnership

A key lesson from this analysis is that the long-term success of PPPs in PHC depends on more than contractual arrangements alone. Partnership sustainability also depends on whether financing systems are perceived by providers as reliable, predictable, and credible. This is particularly important for smaller providers operating with limited financial reserves and narrow operating margins. Strategic purchasing literature similarly emphasizes that provider participation is influenced not only by the existence of contracts but also by the predictability of reimbursement processes, payment systems, and financing arrangements (21, 28).

From this perspective, provider caution should not necessarily be interpreted as opportunistic behavior. Rather, it may reflect rational responses to uncertainty within financing and purchasing systems. Where providers face delays in payment, uncertainty around reimbursement, or broader fiscal pressures, confidence in partnership arrangements may weaken despite the existence of formal contracts (19–21). In PHC systems, these pressures may ultimately affect service continuity, provider participation, and responsiveness to community needs.

The Botswana experience highlights the importance of financing credibility within partnership governance. Recent concerns relating to medicines procurement, purchasing systems, and service delivery management suggest that expanding partnerships alone may be insufficient if broader financing and governance challenges remain unresolved (27, 28). The findings therefore reinforce arguments from the wider strategic purchasing literature that payment reforms are unlikely to succeed in isolation from broader governance and institutional arrangements (30).

More broadly, strategic purchasing is not simply about increasing the number of contracts or providers participating in the system. It is also about whether financing and administrative systems can support predictable cash flow, manageable administrative processes, and sustained provider confidence over time (28, 30). In this sense, financing credibility may be as important to partnership sustainability as the contractual arrangements themselves.

### Accountability beyond contracts: community voice and legitimacy

The evidence suggests that PHC partnerships often place greater emphasis on accountability to ministries, regulators, and purchasers than on accountability to the communities who use services. However, PHC is experienced at community level. Long waiting times, staff attitudes, medicine availability, and provider responsiveness all shape how people experience the health system and whether they continue to trust it (40, 41, 47).

These findings suggest that contractual compliance is necessary but not sufficient for sustaining legitimacy within PHC partnerships. Reporting systems and performance indicators remain important, but they do not always capture how services are experienced by communities. Similar concerns appear in the wider social accountability literature, which suggests that participation mechanisms are most effective when linked to institutions capable of responding to complaints and local concerns in visible and meaningful ways (23, 31).

This issue may be particularly important in PHC settings because care is continuous and relationship-based rather than episodic. Community trust can influence whether people continue seeking care, follow treatment advice, and remain engaged with local services over time. Accountability within PPP arrangements may therefore need to extend beyond contracts, reports, and performance targets alone.

Botswana's PHC reform agenda increasingly emphasizes integrated and people-centered care across public and non-state providers (42, 43). However, translating these principles into everyday practice requires stronger mechanisms for community accountability. Consultative committees, accessible complaints procedures, and clearer feedback pathways may help strengthen communication between communities, providers, and health authorities. From this perspective, accountability in PHC partnerships is not only an administrative issue. It is also closely linked to legitimacy, trust, and the day-to-day relationship between health systems and the communities they serve (23, 31).

### Bridging the national–local divide in PPP implementation

The gap between national policy ambition and local implementation remains an important issue for PHC partnerships. In Botswana, recent policy directions have created more space for decentralized planning, integrated service delivery, and collaboration among different actors. However, the analysis suggests that these ambitions may be constrained by pressures at district and facility levels, where implementation takes place (34, 44, 45).

Botswana is not alone in this regard. The wider decentralization literature shows that responsibilities delegated to local levels are not always accompanied by matching authority, resources, and support (44). As a result, district managers may be expected to implement reforms while working with limited financial discretion, uneven managerial support, and variable organizational capacity. Under these conditions, partnership arrangements that appear manageable at policy level may become more difficult to sustain in practice.

Implementation constraints are therefore not only a matter of policy design. The way partnerships function across districts and institutions may also be shaped by staffing pressures, uneven resource availability, administrative bottlenecks, and the everyday realities of service delivery. Similar concerns have been discussed in the health systems and policy implementation literature, which emphasizes the importance of local context in shaping how reforms are managed in practice (16, 46).

From a PPP perspective, partnerships are sustained not only by national policy commitments, but also by local systems that can manage provider relationships, supervision, reporting, and coordination. Without such systems, partnerships may become uneven and fragmented across districts, even where the overall policy environment appears supportive.

Botswana's PHC restructuring debates provide further support for the need to strengthen coordination between national and local levels of the health sector. Decentralization alone may not improve PPP implementation unless it is accompanied by stable institutional coordination, clear operational roles, and stronger district-level support (34, 45).

### Implications for policy, research, and future PPP design

The study's findings are most applicable in situations where the PHC is financially constrained, and service delivery is fragmented and involves multiple service providers. Since PPP reforms in such situations require more than expansion of contracts, the partnership performance is contingent upon the credibility of financing, the ability to implement, and the coordination of partnerships, as well as the trust of the various stakeholders from the same system.

In this regard, Botswana illustrates the shortcomings of perceiving PPPs from an administrative or procurement standpoint. Analysis shows that partnership performance is influenced by many contracting and policy design elements, including financing pressures, provider behavior, local implementation, and the relationship of accountability established through time (16, 46). Further empirical studies could investigate the relationship of accountability in various districts under diverse fiscal conditions.

The discussion also impacts how PPPs are designed in the context of PHC systems. If partnerships are expanded without financing credibility and local implementation capacity and accountability pressures, arrangements may appear to be functional but are likely to be unsustainable. Thus, PPP governance will be most effective when purchasing systems are reasonably predictable, district systems are sufficiently supported, and community accountability is prioritized more.

Three overarching governance observations emerge from this analysis. First, the financing credibility, particularly with small, reserve constrained providers, has a stronger impact on sustaining the participation of the small providers. Second, the size and type of provider(s) matter. The commercial provider(s) and the community provider(s) will experience the partnership pressures differently, especially in the face of fiscal uncertainty. Third, the incorporation of community issues into the governance and oversight of the PHC

partnerships will enhance the accountability function more effectively than the standard administrative reporting systems.

Overall, these points posit that beyond the contractual design, the PPPs in PHC are influenced by more comprehensive and wider institutional conditions. Although the research case of Botswana is contextual, several of the governance-related challenges examined in this research are observable in other mixed health systems that are under financial and institutional pressures.

## Conclusion

This study examined the governance conditions shaping public–private partnerships (PPPs) within Botswana's primary health care (PHC) system. The findings suggest that partnership performance depends not only on formal contracts or policy commitments, but also on the broader institutional environment within which partnerships operate. In contexts characterized by fiscal pressure, fragmented service delivery, and growing demands on PHC systems, governance arrangements that rely primarily on administrative control may struggle to sustain trust, responsiveness, and long-term collaboration.

The analysis highlights the importance of governance frameworks that support coordination across actors, credible financing arrangements, stronger links between national and local systems, and accountability mechanisms that remain responsive to communities. The findings also suggest that provider differences matter. Smaller providers operating with limited financial and administrative capacity may experience partnership pressures differently from larger and more established organizations.

The Botswana case illustrates governance challenges that are increasingly visible across many mixed health systems in the Global South. Governments are expected to work with a diverse range of service providers while simultaneously safeguarding equity, accountability, and continuity of care under conditions of resource constraint. In such settings, expanding contractual arrangements alone is unlikely to strengthen partnerships if implementation capacity, financing credibility, and institutional coordination remain weak.

The analysis also points to opportunities for strengthening PHC partnerships through digital health systems, strategic purchasing reforms, and more integrated service delivery arrangements. However, these interventions are unlikely to succeed in isolation. Their effectiveness will depend on the governance conditions that support coordination, learning, adaptation, and sustained engagement among public institutions, providers, and communities.

Ultimately, the findings suggest that strengthening PPPs in PHC is not simply a matter of contract management. It is a governance challenge that requires trusted relationships, credible institutions, and implementation systems capable of responding to changing circumstances. Although the analysis is grounded in Botswana's experience, many of the governance pressures identified are likely to be relevant to other mixed health systems facing similar fiscal and institutional constraints.

## Data Availability

The original contributions presented in the study are included in the article/supplementary material, further inquiries can be directed to the corresponding author.
